# *Mucorales* fungi suppress nitric oxide production by macrophages

**DOI:** 10.1128/mbio.02848-23

**Published:** 2023-12-14

**Authors:** Alexandra Y. Soare, Vincent M. Bruno

**Affiliations:** 1 Department of Microbiology and Immunology, University of Maryland School of Medicine, Baltimore, Maryland, USA; 2 Institute of Genome Sciences, University of Maryland School of Medicine, Baltimore, Maryland, USA; The University of Texas Health Science Center at Houston, Houston, Texas, USA

**Keywords:** *Mucorales*, mucormycosis, nitric oxide, macrophages, *Rhizopus*, *Cunninghamella*, *Mucor*

## Abstract

Mucormycosis is classified by the National Institute of Allergy and Infectious Diseases as an emerging disease and is caused by *Mucorales* fungi. Despite the high morbidity and mortality rates associated with the disease, little is known about the host-pathogen interactions that dictate disease progression. The recent surge of mucormycosis cases among COVID-19 patients has thrust the disease and the lack of available treatments into the spotlight. Despite severe fungal angioinvasion and tissue necrosis during infection, clinical observations suggest a lack of pro-inflammatory responses. Understanding immune evasion mechanisms in mucormycosis can help guide potential therapeutic options. In this study, we demonstrate that *Mucorales* fungi can suppress the accumulation of nitric oxide (NO) in lipopolysaccharide- and interferon gamma-stimulated macrophages despite robust expression of the *Nos2* mRNA and inducible nitric oxide synthase protein. This suppressive activity requires fungal viability and direct contact with macrophages and is not due to restricted access to L-arginine substrate. While *Mucorales* fungi appear to be able to remove NO from its environment, it does not account for the full suppression that we observe and suggests that *Mucorales* employs at least two mechanisms. Future experiments will elucidate the mechanisms by which *Mucorales* fungi deplete NO accumulation by macrophages and the implications of this depletion in mucormycosis pathogenesis.

**IMPORTANCE:**

In October 2022, *Mucorales* fungi were listed in the “High Priority Group” on the first-ever list of fungal priority pathogens by the World Health Organization. As the causative agent of mucormycosis, *Mucorales* have become of great clinical and public health importance with growing mucormycosis numbers, notably with the exponential rise of COVID-19-associated mucormycosis cases. Despite the dire need, there are limited therapeutic options to treat mucormycosis. Our research fills in critical gaps of knowledge about how *Mucorales* fungi evade the host immune system. Specifically, we offer evidence that *Mucorales* block nitric oxide production, which is a key mediator and signaling molecule of the mammalian innate immune response to microbial pathogens. Our work offers new insight into immune evasion mechanisms by *Mucorales* fungi.

## OBSERVATION

Pulmonary mucormycosis is caused by inhalation of fungal spores of the order *Mucorales* and is characterized by rapid angioinvasion and tissue necrosis that often results in near 100% fatality (1). The ability of *Mucorales* to avoid killing by phagocytic innate immune cells, such as macrophages, is crucial to establishing infection (2, 3). Activation of these cells initiates a robust antimicrobial response, which includes the release of nitric oxide (NO). NO is a gaseous free-radical molecule that can be produced in macrophages by inducible nitric oxide synthase (iNOS, encoded by *Nos2*), which catalyzes the production of NO from L-arginine in response to pro-inflammatory stimuli, such as pathogen-associated molecular patterns or interferon gamma (IFN-γ). NO is a central mediator in macrophage effector responses owing to its potent antimicrobial properties and signaling role in various immune pathways (4, 5). Several *in vitro* and *in vivo* studies have demonstrated its antimicrobial activity against a wide variety of fungi (6–10), and successful clinical trials demonstrate its potential as a novel antifungal therapeutic (11, 12).

**Fig 1:**
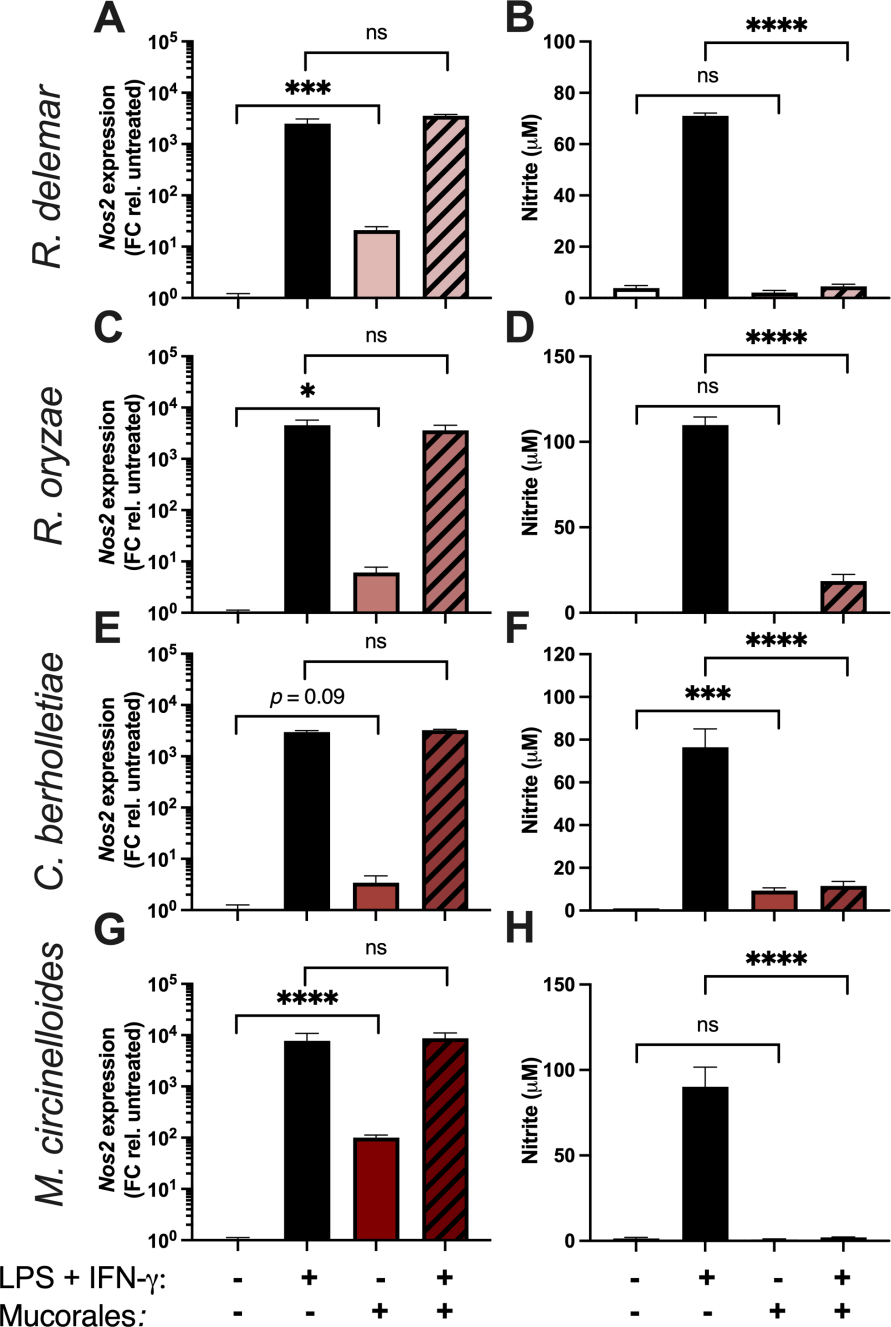
*Mucorales* prevent NO accumulation from activated MH-S macrophages. Monolayers of resting MH-S macrophages were treated with 10 ng/mL LPS and 20 ng/mL IFN-γ, the indicated *Mucorales* species at MOI = 1, or a combination of both treatments. (**A, C, E, G**) After 8 hours, RNA was harvested from the macrophages, and *NOS2* transcript levels were measured by real-time PCR and normalized using primers to β-actin. (**B, D, F, H**) After 24 hours, supernatants were collected and measured for nitrite levels by Greiss assay. In all panels, the data are represented as mean ± SEM of two experiments, each performed in triplicate (*n* = 6; ns, non-significant; ****, *P* < 0.0001; ***, *P* < 0.001 by unpaired, two-tailed student’s *t*-test).

MH-S macrophages infected with *Rhizopus delemar* showed a 10-fold induction of *Nos2* mRNA expression at 8 HPI (Fig. 1A). However, the macrophages did not produce any NO in response to infection with *R. delemar* when supernatants were measured by Greiss assay at 24 HPI (Fig. 1B). Stimulation of MH-S cells with lipopolysaccharide (LPS) and IFN-γ served as a time-matched positive control for *Nos2* expression and NO production (Fig. 1A and B).

We next tested the ability of *R. delemar* to prevent accumulation of NO in response to treatment with LPS and IFN-γ. Addition of *R. delemar* (strain 99-880) conidia to macrophages treated with LPS and IFN-γ almost completely abolished the accumulation of NO in the culture supernatants without reducing the induced expression of the *Nos2* mRNA (Fig. 1A and B). Macrophage death during infection does not account for the full suppression of NO accumulation (Fig. S1). Using the same experimental approach, we obtained similar results with isolates of three additional *Mucorales* species: *Rhizopus oryzae*, *Cunninghamella bertholletiae*, and *Mucor circinelloides* (Fig. 1C through H) indicating that the phenomenon is not strain or species specific. To rule out the possibility that our observations are specific to MH-S cells, we measured *Nos2* expression and NO accumulation following LPS and IFN-γ stimulation and/or infection in the RAW 246.7 cell line as well as primary bone-marrow-derived macrophages (BMDMs). Similar results were obtained in both cases (Fig. S2). The active repression of NO accumulation in macrophages by *Mucorales* suggests that NO may exhibit some anti-*Mucorales* properties. Incubation of *R. delemar* with chemically generated nitric oxide (DETA-NONOate) blocked fungal metabolism with an IC_50_ of approximately 500 µM (Fig. S3A) and reduced fungal viability at concentrations above 150 µM (Fig. S3B).

**Fig 2:**
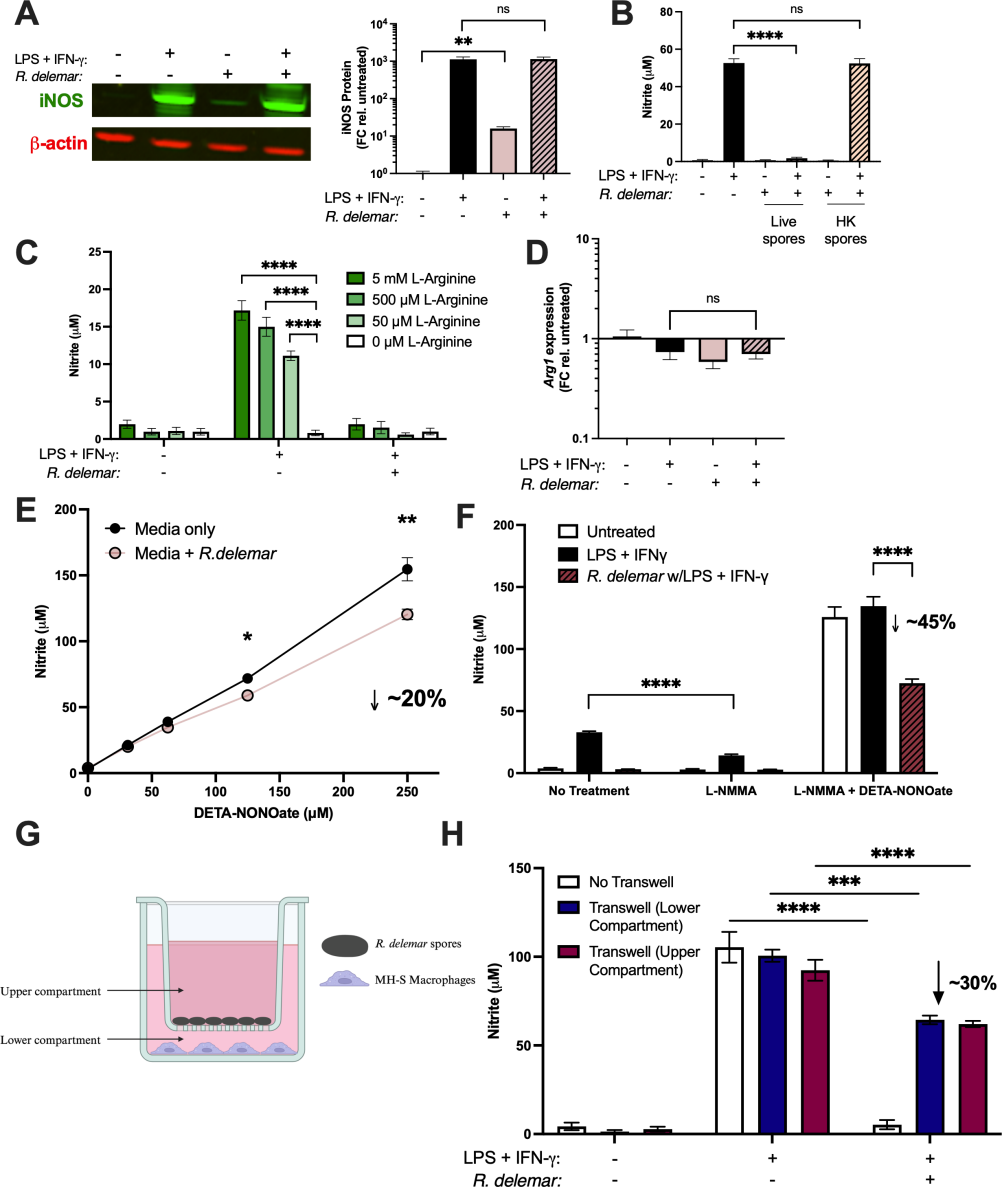
*Mucorales* prevent NO accumulation from activated macrophages by multiple mechanisms. (**A–D**) Monolayers of resting MH-S macrophages were treated with 10 ng/mL LPS and 20 ng/mL IFN-ɣ, the indicated *Mucorales* species at MOI = 1, or a combination of both treatments. (**A**) After 8 hours, protein lysates were harvested from macrophages, and iNOS and β-actin protein levels were quantified by Western Blot. (**B**) Supernatants were collected from macrophages that were treated with live *R. delemar* spores or spores that had been heat-killed at 65°C for 2 hours. Supernatants were collected after 24 hours and measured for nitrite levels by Greiss assay. (**C**) Macrophages were starved in L-arginine-free media for 3 hours before being treated with indicated conditions and L-arginine supplementation. Supernatants were collected after 24 hours and measured for nitrite levels by Greiss assay. (**D**) After 8 hours, RNA was harvested from the macrophages, and *Arg1* transcript levels were measured by real-time PCR and normalized using primers to β-actin. (**E**) Indicated concentrations of DETA-NONOate were incubated in a liquid medium in a six-well plate with or without *R. delemar* (1 × 10^6^ spores per well) for 24 hours. Media were collected and tested for nitrite levels by Greiss assay. (**F**) Monolayers of resting MH-S macrophages were treated with 10 ng/mL LPS and 20 ng/mL IFN-γ, the indicated *Mucorales* strain at MOI = 1, or a combination of both treatments. Macrophages were also treated with L-NMMA alone or L-NMMA with DETA-NONOate. (**G**) Set-up for transwell experiment. (**H**) Monolayers of resting MH-S macrophages were treated with 10 ng/mL LPS and 20 ng/mL IFN-γ in the presence or absence of *R. delemar* at MOI = 1. After 24 hours, supernatants were collected and measured for nitrite levels by Greiss assay. In all panels, the data are represented as mean ± SEM of two experiments, each performed in triplicate (*n* = 6; ns, non-significant; ****, *P* < 0.0001; ***, *P* < 0.001; **, *P* < 0.01; *, *P* < 0.05 by unpaired, two-tailed student’s *t*-test).

LPS- and IFN-γ-treated macrophages cocultured with *R. delemar* showed comparable iNOS protein levels to macrophages that were treated with LPS and IFN-γ alone (Fig. 2A). These results suggest that the inhibition of NO accumulation is not simply the result of reduced iNOS protein levels in the presence of *Mucorales* but rather an inhibition of iNOS enzymatic activity or an active depletion of NO from the culture supernatants. Coculture of LPS- and IFN-γ-treated MH-S cells with heat-killed *R. delemar* spores did not inhibit accumulation of NO in the supernatant (Fig. 2B), indicating that NO repression is due to an active process by *R. delemar* that requires the fungal spores to be viable.

Limited access to L-arginine has been shown to inhibit the ability of iNOS to produce NO during *Helicobacter* infection (13). To determine if *R. delemar* may be limiting accessible L-arginine pools for iNOS, we performed the previous set of experiments in L-arginine-depleted media that were supplemented with increasing amounts of excess L-arginine. In the absence of fungal spores, LPS- and IFN-γ-treated macrophages showed a dose-dependent increase in NO production with increasing concentrations of exogenous L-arginine. As observed above, macrophages stimulated with LPS and INF-γ in the presence of *R. delemar* showed significantly reduced NO production, and this block in NO production was not restored by the addition of excess L-arginine, even at a concentration of 5 mM (Fig. 2C). Additionally, *R. delemar* infection had no effect on the expression of *Arg1,* which encodes an arginase capable of depleting intracellular arginine pools and is induced in context of other fungal infections (Fig. 2D) (7). Taken together, these results suggest that the suppression of NO production by macrophages during *R. delemar* infection is not due to reduced accessibility by iNOS to L-arginine.

We wondered if the lack of NO accumulation was the result of active removal from the media as has been shown for other fungal pathogens (14–16). To this end, we tested the ability of *R. delemar* to remove DETA-NONOate from media in the absence of macrophages. *R. delemar* depleted approximately 20% of the NO from its environment (Fig. 2E). To address the possibility that macrophage contact is necessary to fully activate fungal detoxification systems, we repeated the DETA-NONOate experiments in the presence of MH-S cells that have been treated with L-NMMA, an iNOS inhibitor, to ensure that none of the NO was derived from the macrophage iNOS activity (17). As expected, MH-S cells treated with L-NMMA produced less NO in response to LPS and IFN-γ stimulation, which was rescued upon addition of DETA-NONOate (Fig. 2F). In this experimental format, *R. delemar* was able to deplete 45% of the NO from the system. These data suggest that *R. delemar* can partially remove NO from its environment but it is not responsible for the complete ablation of NO during *in vitro* infection.

We next tested if *R. delemar* needed to be in direct contact with macrophages to suppress the accumulation of NO. To this end, we repeated the experiments with each cell type on opposite sides of a Transwell membrane that would allow for diffusion of small molecules but prevent contact with *R. delemar* (Fig. 2G). As expected, NO levels from stimulated macrophages with *R. delemar* on the same side of the Transwell were dramatically lower compared to macrophage cultures without *R. delemar* (Fig. 2H). The separation of contact between the stimulated macrophages and *R. delemar* by the Transwell membrane restored NO accumulation to ~70% of the positive control (LPS and IFN-γ, no fungus, no Transwell; Fig. 2H), indicating that direct contact is necessary for the full suppressive activity by *R. delemar*. The ~33% reduction in NO accumulation, compared to control macrophages, is likely to be the result of NO detoxification that we observed (Fig. 2E and F).

### DISCUSSION

In this observation, we uncover a new phenomenon in which *Mucorales* fungi suppress a critical innate effector function in activated macrophages and propose that *Mucorales* fungi exert this phenomenon by at least two different mechanisms: one that involves the active removal of NO from the media and another that requires direct contact with macrophages. Our findings have implications for co-infections or co-occurrence of *Mucorales* with other pathogens, such as Gram-negative bacteria (18–20), SARS-CoV-2, and other fungi (21). Specifically, by depleting NO, *Mucorales* may prevent macrophages from appropriately responding to these co-infecting pathogens.

Other fungal pathogens including *Candida albicans*, *Coccidiodes* spp., *Blastomyces dermatitidis*, and *Cryptococcus neoformans* have also been shown to inhibit production of NO in macrophages *in vitro* (22–25). In all cases, the fungal molecule responsible for the inhibition has not been identified. There is much that remains unknown as to how NO exerts its antifungal effects. In addition to directly killing fungi, NO has been shown to act as a signaling molecule in the development of fungi (26). Whether NO exerts these effects on *Mucorales* remains unknown. Additionally, NO has been reported to govern the “glycolytic switch” for pro-inflammatory macrophage phenotype (27, 28). By preventing NO production in activated macrophages, *Mucorales* fungi may not only prevent the production of an antimicrobial molecule but also prevent important, NO-dependent signaling required for macrophages to control fungal infection. At the moment, the molecular basis by which NO accumulation is blocked by *Mucorales* remains unknown. Further experiments are required to elucidate these mechanisms and to determine the physiological consequences of NO depletion during mucormycosis.

## SUPPLEMENTAL MATERIAL

Figure S1 mbio.02848-23-s0001.pdf LDH release from *R. delemar*-infected MH-S macrophages.

Figure S2 mbio.02848-23-s0002.pdf *R. delemar* depletes NO in multiple activated macrophage models.

Figure S3 mbio.02848-23-s0003.pdf *R. delemar* growth is inhibited by chemically generated nitric oxide.

Supplemental Methods mbio.02848-23-s0004.docx Descriptions of all methods used in this paper and legends for the supplemental figures.

ASM does not own the copyrights to Supplemental Material that may be linked to, or accessed through, an article. The authors have granted ASM a non-exclusive, world-wide license to publish the Supplemental Material files. Please contact the corresponding author directly for reuse.
