# Supplementary material for: Mucorales fungi suppress nitric oxide production by macrophages
Source: mBio. 2023 Dec 14;15(1):e02848-23. doi: 10.1128/mbio.02848-23 (PMC10790689; doi:10.1128/mbio.02848-23)
Supplement: Figure S1 — LDH release from R. delemar-infected MH-S macrophages. [file mbio.02848-23-s0001.pdf]

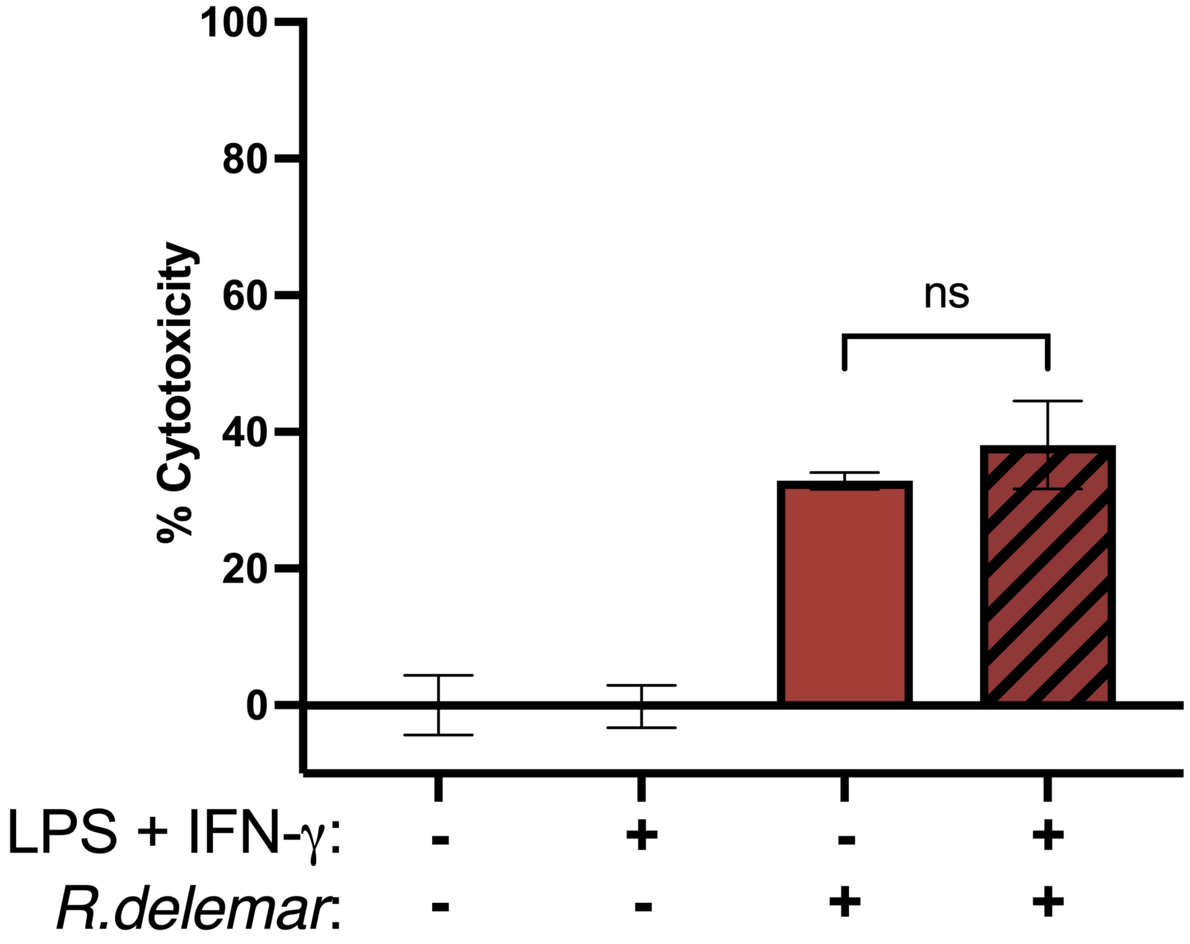

Supplementary Figure 1. LDH-release from *R. delemar*-infected MH-S macrophages. Monolayers of MH-S macrophages were treated with 10 ng/mL LPS and 20 ng/mL IFN-γ, *R. delemar* at an MOI = 1, or a combination of both treatments. After 24 hours, extracellular lactate dehydrogenase (LDH) was quantified as a measurement of MH-S cell death. Data is represented as mean ± SEM (n = 3; ns, non-significant by unpaired, two-tailed student's T-test).
