# Supplementary material for: Mucorales fungi suppress nitric oxide production by macrophages
Source: mBio. 2023 Dec 14;15(1):e02848-23. doi: 10.1128/mbio.02848-23 (PMC10790689; doi:10.1128/mbio.02848-23)
Supplement: Figure S2 — R. delemar depletes NO in multiple activated macrophage models. [file mbio.02848-23-s0002.pdf]

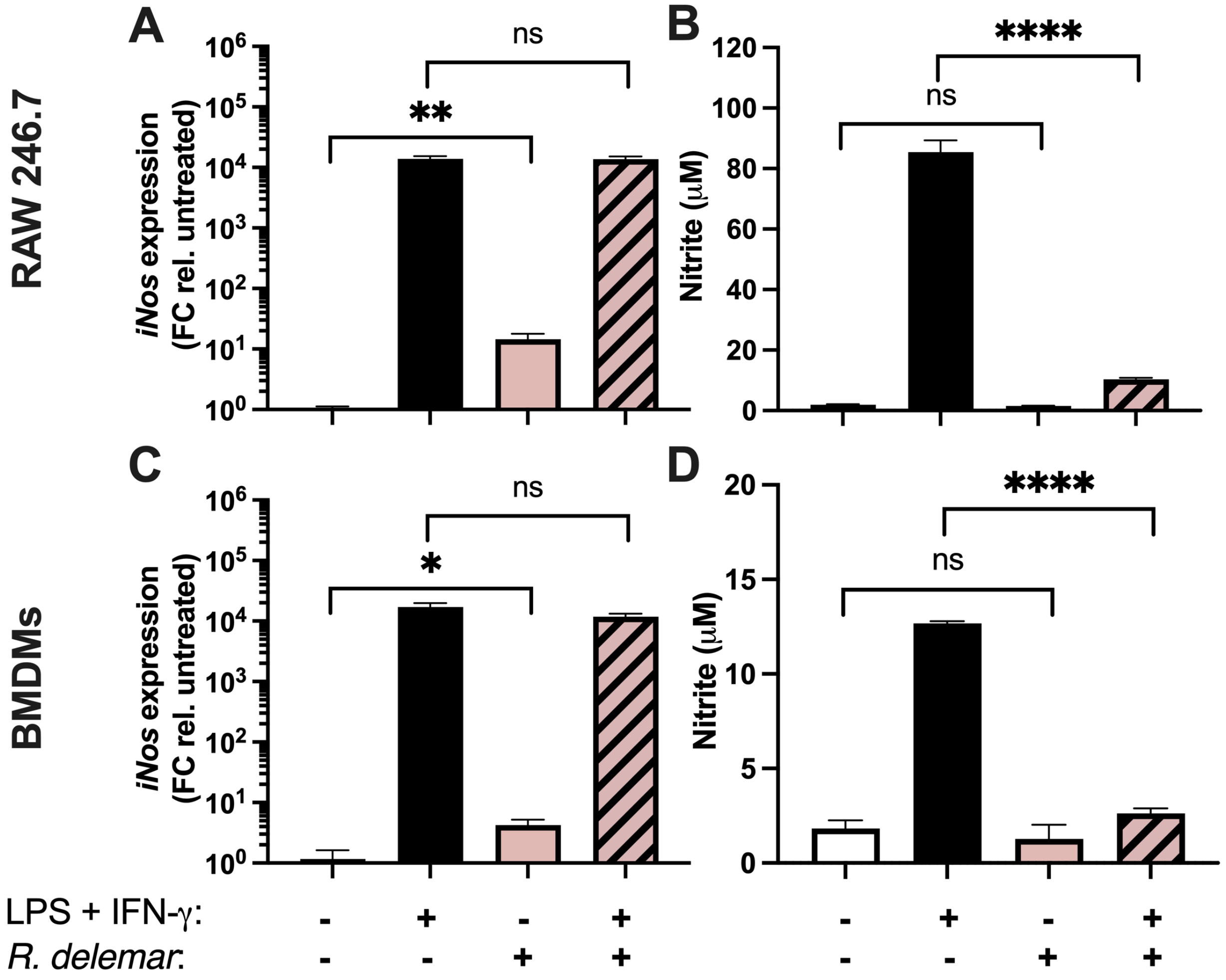

Supplementary Figure 2. *R. delemar* depletes NO in multiple activated macrophage models. Monolayers of indicated macrophage models were treated with 10 ng/mL LPS and 20 ng/mL IFN-γ, *R. delemar* at an MOI = 1, or a combination of both treatments. (A, C) After 8 hours, RNA was harvested from the macrophages and *NOS2* transcript levels were measured by real-time PCR and normalized using primers to β-actin. (B, D) After 24 hours, supernatants were collected and measured for nitrite levels by Greiss assay. In all panels, the data are represented as mean ± SEM of 2 experiments, each performed in triplicate (n = 6; ns, non-significant; ****, $p < 0.0001$ by unpaired, two-tailed student's T-test).
