# Supplementary material for: Mucorales fungi suppress nitric oxide production by macrophages
Source: mBio. 2023 Dec 14;15(1):e02848-23. doi: 10.1128/mbio.02848-23 (PMC10790689; doi:10.1128/mbio.02848-23)
Supplement: Figure S3 — R. delemar growth is inhibited by chemically generated nitric oxide. [file mbio.02848-23-s0003.pdf]

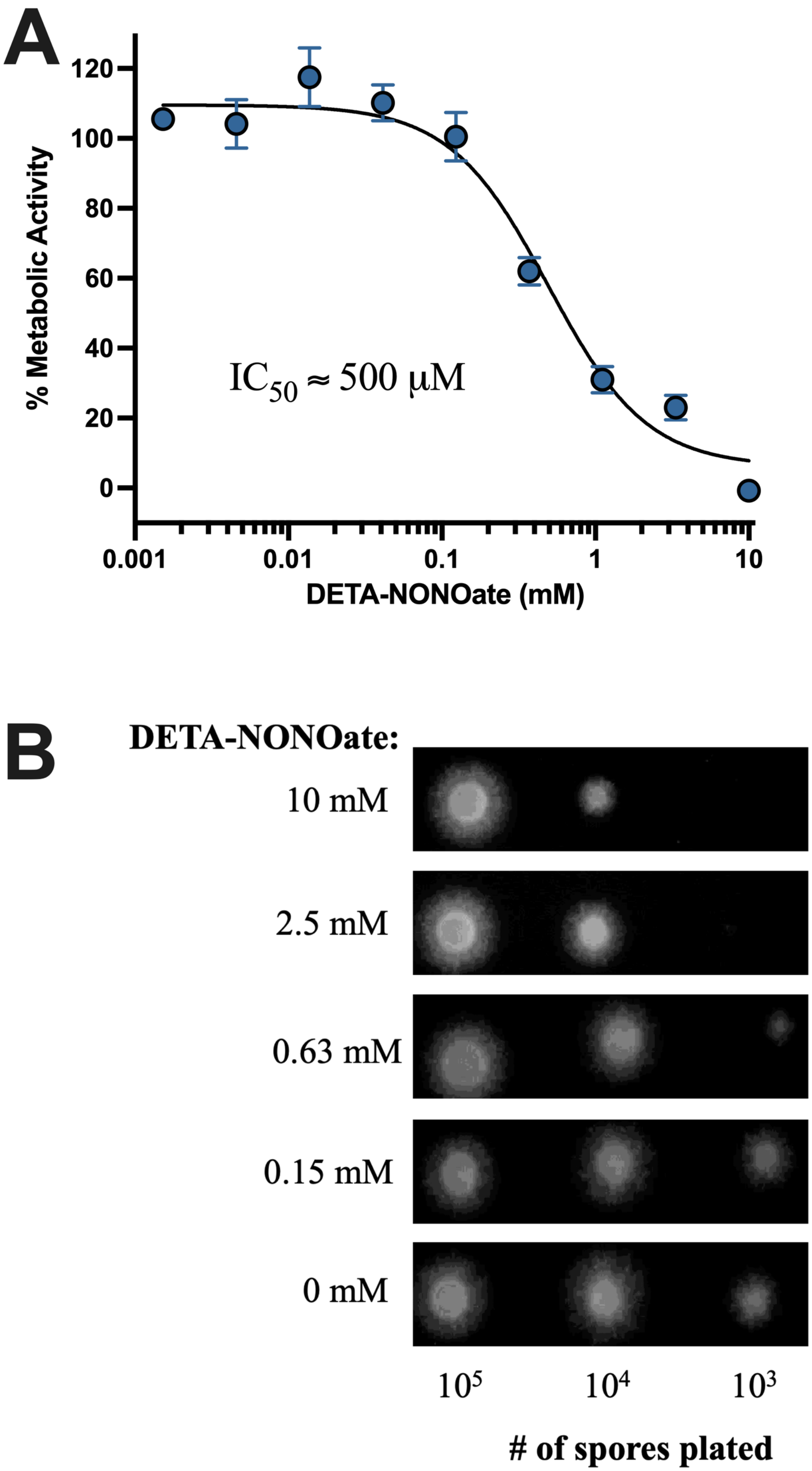

A
120
100
80
60
40
20
0
% Metabolic Activity
IC50 ≈ 500 µM
0.001
0.01
0.1
1
10
DETA-NONOate (mM)
B
DETA-NONOate:
10 mM
2.5 mM
0.63 mM
0.15 mM
0 mM
10^5
10^4
10^3
# of spores plated

Supplementary Figure 3: *R. delemar* growth is inhibited by chemically-generated nitric oxide. (A) *R. delemar* spores were treated with DETA-NONOate for 24 hours in a 96-well plate. CellTiter 96® AQ_{ueous} One Solution Reagent was added for 2 hours then absorbance was read at 490 nm. OD_{490} values were normalized to untreated spores and a nonlinear fit (sigmodal, 4 parameter logistic repression) was generated to determine IC_{50}. The data is represented as mean ± SEM of 3 experiments, each performed in duplicate (*n* = 6). (B) Indicated amount of *R. delemar* spores were incubated with varying concentrations of DETA-NONOate. Spores were plated onto DRBC plates then imaged for visualization after 12-16 hours**.**
