# Supplementary material for: Mucorales fungi suppress nitric oxide production by macrophages
Source: mBio. 2023 Dec 14;15(1):e02848-23. doi: 10.1128/mbio.02848-23 (PMC10790689; doi:10.1128/mbio.02848-23)
Supplement: Supplemental Methods — Descriptions of all methods used in this paper and legends for the supplemental figures. [file mbio.02848-23-s0004.docx]

**Supplemental Information**

**MATERIALS AND METHODS**

**Macrophage cells**

MH-S macrophages (ATCC, #CRL-2019) were grown in RPMI 1640 media (ATCC, #30-2001) supplemented with 10% heat-inactivated fetal bovine serum (FBS; Sigma, #M3148), 1% penicillin-streptavidin (Gibco, #15140-122), and 0.05 mM 2-mercaptoethanol (Sigma, #M3148), hereby known as “Complete Media”.

RAW 246.7 macrophages were generously donated by Christen Grassel and were maintained in Dulbecco’s Modified Eagle’s Medium (DMEM; Corning #10-017-CV) with 10% FBS, 1% penicillin-streptavidin, and 1 mM sodium pyruvate (Sigma, #F4135).

Bone-marrow derived macrophages (BMDMs) were isolated from femurs and tibia of male 6-10 week old C57BL/6 mice and generously donated by the Joao Pedra laboratory. BMDMs were maintained in DMEM media with 10% FBS, 1% penicillin-streptavidin, and 30% L929 cell condition medium as a source of macrophage colony-stimulating factor. Cells were cultured for 5-6 days before infections.

**Isolation of fungal spores**

*Rhizopus delemar* 99-880, *Rhizopus oryzae* 99-892*, Cunninghamella berholletiae* 175*, and Mucor circinelloides* CBS 277.49 were grown on peptone-dextrose agar (PDA) plates for 3-5 days at 37$^{\circ}$C. Spores were collected in endotoxin-free Dulbecco’s phosphate buffer (DPBS, Invitrogen #ING-14190144) and passed through a cell-strainer to separate spores from mycelium. Spores were washed twice in DPBS through centrifugation at 3,000 rpm for 5 minutes and counted with a hemocytometer to prepare for final inoculum. Where indicated, spores were heat-killed by incubation at 65°C for 2 hours and verified by monitoring growth for 2 days after streaking onto a PDA plate.

**Macrophage-Mucorales cocultures**

Indicated macrophages were plated in tissue culture plates at 0.5 x 10^6^ cells in 6-well plates or 0.8 x 10^6^ cells in 60 mm plates for 24 hours at 37°C, 5% CO_2_. Macrophages were presumed to have doubled in number and treated with 10 ng/mL LPS and 20 ng/mL IFN-$\gamma$ in the presence or absence of indicated fungal spores (MOI = 1). RNA and protein lysates were collected at 8 hours post-infection (HPI) for RT-qPCR and Western Blots, respectively. Supernatants were harvested at 24 HPI and stored at -80$^{\circ}$C until future use.

Where indicated, 22 mm transwells with 0.4 μm pore polyester membrane inserts (Costar #3450) were used to separate the fungus from the macrophages in 6-well plates.

## **Measurement of *R. delemar*-induced damage of macrophages**

*R. delemar*-induced damage of MH-S macrophages was measured by CyQUANT™ LDH Cytotoxicity Assay Kit (Invitrogen #C20300) according to manufacturer’s instructions. Lactate dehydrogenase (LDH) is a cytosolic enzyme that is released into cell culture medium upon cell membrane damage (*1*). Briefly, 2 x 10^4^ MH-S macrophages were grown in 96-well plates for 24 hours in Complete Media. At time of infection, media was replaced and macrophages were treated with 10 ng/mL LPS and 20 ng/mL IFN-$\gamma$, fungal spores (MOI = 1), or fungal spores in combination with LPS and IFN-$\gamma$. After 24 hours of incubation, 10 μL of Lysis Buffer was added to designated wells serving as “host cell maximum release control”. 10 μL of sterile ultrapure water was added to all other walls and the plate was incubated for 45 minutes. At the end of the incubation 50 μL of cell culture supernatant was added to new 96-well plate and incubated with 50 μL of Reaction Mixture. Plate was incubated at room temperature for 30 minutes protected from light. To stop reaction, 50 μL of Stop Solution was added and absorbance was measured at 490 nm and 680 nm. To determine LDH activity, 680-nm absorbance value (background signal from instrument) was subtracted from 490-nm absorbance value. LDH release was calculated as follows: % cytotoxicity = [(experimental release – fungal cell spontaneous control – host cell spontaneous control)/(host cell maximum release control – host cell spontaneous control)] × 100.

**RNA isolation and RT-qPCR**

At indicated times, total mRNA was isolated from macrophages using TRIzol (Invitrogen, #15596026) and PureLink RNA Mini Kit (Invitrogen, #12183020) according to manufacturer’s instructions. RNA was quantified by Agilent RNA ScreenTape and Agilent 4200 TapeStation System (Agilent, #G2991BA). RNA was treated with DNase and converted to cDNA with Thermo Scientific^TM^ Maxima^TM^ H Minus cDNA Synthesis Master Mix (Thermo Scientific, #M1681). Real-time qPCR was performed using KAPA SYBR^®^ FAST qPCR Master Mix (2X) Kit (Kapa Biosystems, #K40389). The following protocol was used on a C1000 Touch Thermal Cycler (Bio-Rad): 3 min at 95$℃$, followed by 40 cycles of 95$℃$ for 3 sec and 60$℃$ for 40 sec, followed by melting curve analysis. Transcript levels for targets were compared to $\beta$-actin and fold-change in expression was calculated by the 2^-^$\Delta\Delta$^Ct^ method (*2*). The following primers were ordered from Integrated DNA Technologies: *Nos2* (Forward, 5’-ACATCGACCCGTCCACAGTAT-3’; Reverse, 5’-CAGAGGGGTAGGCTTGTCTC-3’), *Arg1* (Forward, 5’-ACAGCAAAGCAGACAGAACTA-3’; Reverse, 5’-GAAAGGAACTGCTGGGATACA-3’), and $\beta$*-actin* (Forward, 5’-GACGTTGACATCCGTAAAGACC-3’; Reverse, 5’-GCAGTAATCTCCTTCTGCATCC-3’).

**Immunoblot of iNOS *in vitro***

MH-S cells were grown in and treated in manner described above. At 8 HPI, cells were washed with ice-cold 1X endotoxin-free DPBS with 1 mM Na_3_VO_4_. Cell lysis buffer [1X Cell Lysis Buffer (Cell Signaling, #9803), 1 mM PMSF (Thermo Scientific, #36978), 1% Phosphatase Inhibitors (Sigma, #P5726, P0044) in Molecular Grade Water] was added to monolayer and incubated on ice for 5 minutes. The cells were scraped from surface, transferred to tubes and sonicated three times at 40% maximum amplitude for 6 seconds. Debris was pelleted at 4,000 rpm for 10 minutes and supernatants were transferred to fresh tubes. Protein concentrations were quantified by Pierce^TM^ BCA protein Assay (Thermo Scientific, #23227) and 10 ug were separated by NuPAGE^TM^ 4-12%, Bis-Tris gels (Thermo Fisher, #WG1402BOX). Proteins were transferred to PVDF membranes, which were blocked for 1 hour in Intercept Blocking Buffer (LI-COR, #927-60001), then probed with a 1:1,000 dilution of anti-iNOS antibody (Abcam, #ab178045) and $\beta$-actin (Cell Signaling, #3700) overnight at 4$℃$. The membranes were washed 3 times with TBS-T and incubated for 1 hour with 1:15,000 dilution of secondary antibodies (LI-COR, #926-32213 and #926-68072). The membranes were washed 3 times with TBS-T then bands were visualized and quantified with an Odyssey CLx (LI-COR).

***L*-Arginine depletion and exogenous addition**

5 x 10^4^ MH-S macrophages were seeded onto 24-well plates and cultured at 37°C, 5% CO_2_ for 24 hours. Three hours before infection, monolayers were washed with DPBS and incubated in RPMI 1640 media for SILAC (Thermo, #88365) supplemented with 0.4 g/L *L*-lysine (Sigma, #L-5001), 10% dialyzed fetal bovine serum (Thermo, #A3382001), 10 mM HEPES (Thermo, #15630080) and 1% penicillin-streptavidin, hereby known as “*L*-arginine free media”. After starvation, medium was removed and fresh *L*-arginine free media was added with indicated conditions (10 ng/mL LPS and 20 ng/mL IFN-$\gamma$; *R. delemar* at MOI = 1) and supplemented *L*-Arginine (Sigma, #1111009) concentrations as indicated. After 24 hours, supernatants were harvested and analyzed for nitrite concentrations.

**Detoxification Experiments**

For macrophage-free detoxification experiments, varying concentrations of NO donor DETA-NONOate (abcam #ab144627) were incubated in Complete Medium in a 6-well plate. Each concentration was grown either in the presence or absence of 1 x 10^6^ *R. delemar* spores per well. After 24 hours, supernatants were harvested and analyzed for nitrite concentrations.

For macrophage co-culture detoxification assays, MH-S cells were pre-treated with 100 $\mu$M iNOS inhibitor L-NG-monomethyl arginine citrate (L-NMMA; Cayman Chemical, #80200) as previously described (*3*). After 1 hour, cells were treated with 125 $\mu$M DETA NONOate with indicated conditions. Supernatants were collected at 24 hours for further analysis. A fresh tube of DETA-NONOate was reconstituted for each experiment.

**Nitrite determination**

At indicated times, supernatants were harvested from cultures and frozen at -80$℃$ until used for nitrite determination as an indicator of NO production via a Greiss Reagent kit (Molecular Probes #G-7921). Samples were spun for 15 minutes at 14,000 x g with 10,000 MW cut-off filters for deproteination and diluted before mixed with equal amounts of Component A (*N*-(1-naphthyl-ehtylenediamine) dihydrochloride) and Component B (Sulfanilic acid). After 30 minutes incubation in the dark, absorbance at 548 nm was measured and nitrite concentrations were extrapolated from standard curve of sodium nitrite.

## **Sensitivity of *R. delemar* to nitric oxide**

To quantify *R. delemar* growth in the presence of nitric oxide, a MTS assay was performed to measure *R. delemar* viability. In this assay, a tetrazolium inner salt (Owen’s Reagent) is reduced by NAD(P)H-dependent dehydrogenase enzymes in viable cells to formazan, which can be quantified at 490 nm and indicates cellular metabolic activity (*223*). This assay has been used as a measurement for fungal metabolism and a proxy for fungal growth (*224, 225*). 1 x 10^4^ *R. delemar* spores were plated in a 96-well plate with varying concentrations of DETA-NONOate in 200 $\mu$L. After 24 hours, 40 $\mu$L of CellTiter 96^®^ AQ_ueous_ One Solution Reagent (Promega, #G3580) was added to all wells and incubated in humidified 37$^{\circ}$C, 5% CO_2_ incubator for 1-2 hours. Following incubation, 50 $\mu$L were transferred to a fresh plate and absorbance was measured at 490 nm. Metabolic activity was calculated as followed: % metabolic activity = [(corrected OD_490_ of fungi / corrected OD_490_ of non-treated fungi) × 100].

For visualization of fungal growth, 10-fold dilutions of *R. delemar* spores were incubated with varying concentrations of DETA-NONOate in a 96-well plate in a humidified 37$^{\circ}$C, 5% CO_2_ incubator. After 3 hours, spores were spotted onto Dichloran Rose-Bengal Chloramphenicol (DRBC) agar plates. The dichloran in DRBC limits filamentation and allow for visualization of fungicidal effects of compounds on filamentous fungi, which normally overgrow on agar plates (*226*). The plates were incubated at 37$^{\circ}$C for 12-16 hour before imaging.

**Statistical analysis.**

Graphs were generated with Prism 9. Data are expressed as mean $\pm$ standard error of mean (SEM). Differences between groups were analyzed for statistical significance using unpaired, two-tailed Student’s *t* tests. A *P* value of less than 0.05 was considered statistically significant.

**SUPPLEMENTAL FIGURES LEGENDS**

**Supplementary Figure 1. LDH-release from *R. delemar*-infected MH-S macrophages.** Monolayers of MH-S macrophages were treated with 10 ng/mL LPS and 20 ng/mL IFN-$\gamma$, *R. delemar* at an MOI = 1, or a combination of both treatments. After 24 hours, extracellular lactate dehydrogenase (LDH) was quantified as a measurement of MH-S cell death. Data is represented as mean ± SEM (*n* = 3; ns, non-significant by unpaired, two-tailed student’s T-test).

**Supplementary Figure 2. *R. delemar* depletes NO in multiple activated macrophage models.** Monolayers of indicated macrophage models were treated with 10 ng/mL LPS and 20 ng/mL IFN-$\gamma$, *R. delemar* at an MOI = 1, or a combination of both treatments. (A, C) After 8 hours, RNA was harvested from the macrophages and *NOS2* transcript levels were measured by real-time PCR and normalized using primers to β-actin. (B, D) After 24 hours, supernatants were collected and measured for nitrite levels by Greiss assay. In all panels, the data are represented as mean ± SEM of 2 experiments, each performed in triplicate (*n* = 6; ns, non-significant; ****, *p* < 0.0001 by unpaired, two-tailed student’s T-test).

**Supplementary Figure 3: *R. delemar* growth is inhibited by chemically-generated nitric oxide.** (A) *R. delemar* spores were treated with DETA-NONOate for 24 hours in a 96-well plate. CellTiter 96® AQ_ueous_ One Solution Reagent was added for 2 hours then absorbance was read at 490 nm. OD_490_ values were normalized to untreated spores and a nonlinear fit (sigmodal, 4 parameter logistic repression) was generated to determine IC_50_. The data is represented as mean ± SEM of 3 experiments, each performed in duplicate (*n* = 6). (B) Indicated amount of *R. delemar* spores were incubated with varying concentrations of DETA-NONOate. Spores were plated onto DRBC plates then imaged for visualization after 12-16 hours**.**

**REFERENCES**

1. P. Kumar, A. Nagarajan, P. D. Uchil, Analysis of cell viability by the lactate dehydrogenase assay. *Cold Spring Harb. Protoc* **2018**, 465-468 (2018).

2. K. J. Livak, T. D. Schmittgen, Analysis of relative gene expression data using real-time quantitative PCR and the 2(-Delta Delta C(T)) Method. *Methods* **25**, 402-408 (2001).

3. N. M. Rocco, J. C. Carmen, B. S. Klein, Blastomyces dermatitidis yeast cells inhibit nitric oxide production by alveolar macrophage inducible nitric oxide synthase. *Infection and immunity* **79**, 2385-2395 (2011).
